# Microstructure and Wear Resistance of Si-TC4 Composite Coatings by High-Speed Wire-Powder Laser Cladding

**DOI:** 10.3390/ma17051126

**Published:** 2024-02-29

**Authors:** Boxuan Men, Shenzhen Sun, Chunyang Hu, Qi Zhang, Bin Han

**Affiliations:** 1 School of Materials Science and Engineering, China University of Petroleum (East China), Qingdao 266580, China; boxuan_men@u.nus.edu (B.M.); 17854227426@163.com (C.H.); zq18353248786@163.com (Q.Z.); 2 Anhui Jianghuai Automobile Group LTD Technical Center, Hefei 230022, China; 17863962286@163.com

**Keywords:** high-speed wire-powder laser cladding, Si-TC4 composite coatings, microstructure, wear resistance

## Abstract

The hardness and wear resistance of the surface of TC4 titanium alloy, which is widely used in aerospace and other fields, need to be improved urgently. Considering the economy, environmental friendliness, and high efficiency, Si-reinforced Ti-based composite coatings were deposited on the TC4 surface by the high-speed wire-powder laser cladding method, which combines the paraxial feeding of TC4 wires with the coaxial feeding of Si powders. The microstructures and wear resistance of the coatings were analyzed using X-ray diffraction (XRD), scanning electron microscopy (SEM), Vickers hardness tester, and friction and wear tester. The results indicate that the primary composition of the coating consisted of α-Ti and Ti_5_Si_3_. The microstructure of the coating underwent a notable transformation process from dendritic to petal, bar, and block shapes as the powder feeding speed increased. The hardness of the composite coatings increased with the increasing Si powder feeding rate, and the average hardness of the composite coating was 909HV_0.2_ when the feeding rate reached 13.53 g/min. The enhancement of the microhardness of the coatings can be attributed primarily to the reinforcing effect of the second phase generated by Ti_5_Si_3_ in various forms within the coatings. As the powder feeding speed increased, the wear resistance initially improved before deteriorating. The optimal wear resistance of the coating was achieved at a powder feeding rate of 6.88 g/min (wear loss of 2.55 mg and friction coefficient of 0.12). The main wear mechanism for coatings was abrasive wear.

## 1. Introduction

Titanium alloys have been widely used in aerospace [1], automotive [2], and biomedical [3] fields, due to their high specific strength [4] and good corrosion resistance [5]. However, the low shear strength and poor wear resistance of titanium alloy surfaces [6,7] shorten their service life, so improving the wear resistance of titanium alloy surfaces is of great significance for the application of titanium alloys [8,9,10,11]. Surface modification techniques, such as laser cladding [7,9,12], are widely used to improve the surface properties of metals. Laser cladding technology has become an important modification method for metal surfaces, due to its high efficiency, low dilution rate, and low thermal impact on the substrate [13]. In recent years, high-speed laser cladding has been widely studied because of its advantages in terms of efficiency, dilution rate control, and performance [14].

Currently, the materials used in laser cladding are mainly metal powders, and the feeding methods include pre-positioned powder spreading [15,16] and coaxial powder feeding [17,18]. The powder feeding, laser cladding laser energy absorption rate is high, easy for automating the control, but the powder utilization rate is not high, and the quality of the powder requirements are high. Laser wire melting [19] uses metal wire as the material, and compared with powder as raw material, it has the advantages of high processing efficiency, high material utilization, a large degree of freedom in production, good surface-forming quality, high production efficiency, no powder pollution, and so on, and it has been widely studied [20,21,22,23]. In addition to the laser melting of pure metal wires, some researchers have also carried out laser melting studies on core-spun wires [24]. The process of laser melting of wire at the same time, according to the need to add adjustable content of the powder, is a new research idea. Wire-powder synergistic laser cladding combines the advantages of wire-feeding laser cladding and powder feeding laser cladding [25], and at the same time, it reduces the oxidation problem of titanium alloys in the cladding process to a certain extent. At present, researchers have carried out a lot of work on the direct addition of hard phases, such as WC [26], La_2_O_3_, TiB, and TiC particles [27], in wire-powder synergistic laser cladding. Although the direct addition of hard phases to titanium-based alloys can significantly improve their hardness and properties, the differences in thermophysical properties, etc., are prone to cause problems such as cracks and poor bonding.

Compared to titanium matrix composites with the introduction of non-in situ ceramic particles, particle-reinforced near-titanium alloys prepared by the in situ method have the advantages of high wettability and interfacial bonding strength, low contamination, controllable size of the generated reinforcing phases, and low preparation cost [28]. Numerous studies have shown that Ti5Si3 and other in situ precipitated intermetallic compound-reinforced phases have the advantages of low density, high melting point, and excellent resistance to high-temperature oxidation and high-temperature stability [29], which can significantly improve the microhardness and wear resistance of the coatings [30]. The Ti_3_SiC_2_/Ti_5_Si_3_/TiC/Ni25 composite coatings prepared by Yan Hua [31] using laser fusion cladding on TC4 surfaces have excellent microhardness and wear resistance. Zhou Zhongyan [32] prepared Ti_5_Si_3_/Al_3_Ni_2_-enhanced composite coatings on the surface of TC4, and the microhardness, wear resistance, and oxidation resistance of the coatings were significantly improved. Cui Jianxiao [33] prepared an in situ particle-enhanced Ti_x_Al/Ti_5_Si_3_ composite coating on a Ti6Al4V alloy by laser melting, which significantly improved the wear resistance of titanium alloy. Nevertheless, the existing procedures for obtaining in situ Ti_5_Si_3_ and other intermetallic compounds are relatively complex. The combination of Si and Ti elements using techniques like wire–powder synergy and the in situ generation of Ti–Si intermetallic compounds holds significant research value [34,35].

The study presents a method of preparing a composite coating by concurrently laser cladding TC4 wire and Si powder particles onto the surface of TC4 in order to enhance its hardness and wear resistance. The study also investigates the influence of Si content on the evolution of the composite coating’s structure and its wear resistance. Without changing the basic properties of the base material, this study can economically and efficiently prepare the second phase in situ reinforced modified coating with similar thermophysical properties, good metallurgical bonding, and significantly improved performance on the TC4 surface, which creates a new technology of high-efficiency reinforcement of the TC4 surface, fills in the gaps in the field of high-speed wire-powder laser cladding on the surface of TC4, and has strong significance in scientific research and the value of engineering application.

## 2. Experimental Procedures

### 2.1. Coatings Preparation

The substrates for laser cladding were the 100 mm × 50 mm × 10 mm TC4 plates that were smoothed and cleaned. The cladding material was TC4 wire with a diameter of 1.2 mm and pure Si powder with a purity of 99.5% and a particle size of 30–45 μm. The chemical composition of the TC4 wire used for laser cladding is shown in Table 1.

**Figure 1:**
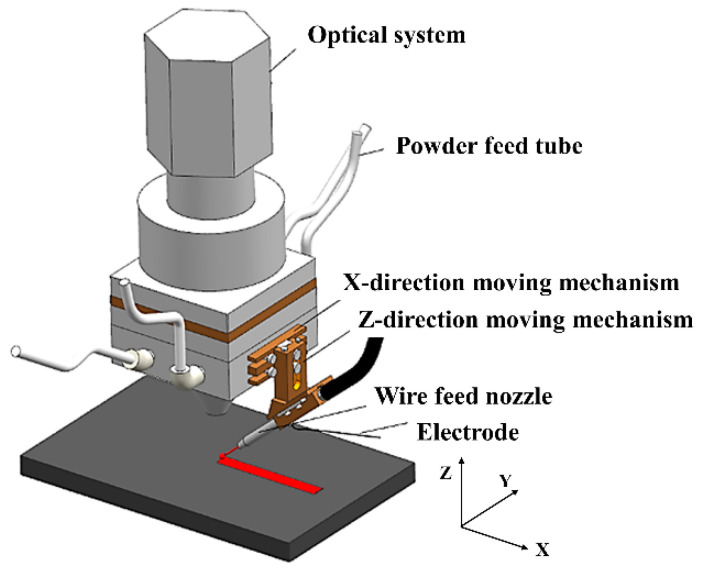
Schematic diagram of the high-speed wire-powder laser cladding head.

The laser cladding experiments were carried out using the high-speed wire-powder laser cladding equipment (ProLC-3000MT, Shaanxi Tyontech, Xi’an, China) protected by 99.99% argon gas. To satisfy the synergistic feeding of wire and powder into the melt pool, the laser cladding head was modified, as shown in Figure 1. The TC4 wire, which was pre-heated by the welder, was fed in the side axis at an angle of 40° to the laser beam. The Si powders, on the other hand, were added to the melt pool coaxially by four uniformly distributed powder feeding tubes after being transported by high-purity argon gas at different powder disk speeds in the powder feeder. Well-formed and defect-free fused cladding layers of titanium matrix composites were prepared under the laser cladding process in Table 2 and different powder feeding speeds (3.35 g/min, 4.48 g/min, 6.88 g/min, 8.43 g/min, 11.06 g/min, and 13.53 g/min).

### 2.2. Microstructures Characterization

After laser cladding, the coatings underwent dye penetrant inspection, wire cutting, grinding, and polishing. Subsequently, the specimens were corroded using an etchant with a composition ratio of HNO_3_:HF:H_2_O = 3:2:95. The microstructure of the coating cross-section and the micro-zone chemical composition were analyzed using a scanning electron microscope (JSM-7200F, JEOL, Tokyo, Japan) equipped with EDS. The phase composition of the resulting coatings was analyzed using an X-ray diffractometer (X’Pert PRO MPD, Panalytical, Almelo, The Netherlands) with Cu-Kα radiation over a scanning range of 20° to 100°and a scanning speed of 18°/min.

### 2.3. Wear Resistance Testing

**Figure 2:**
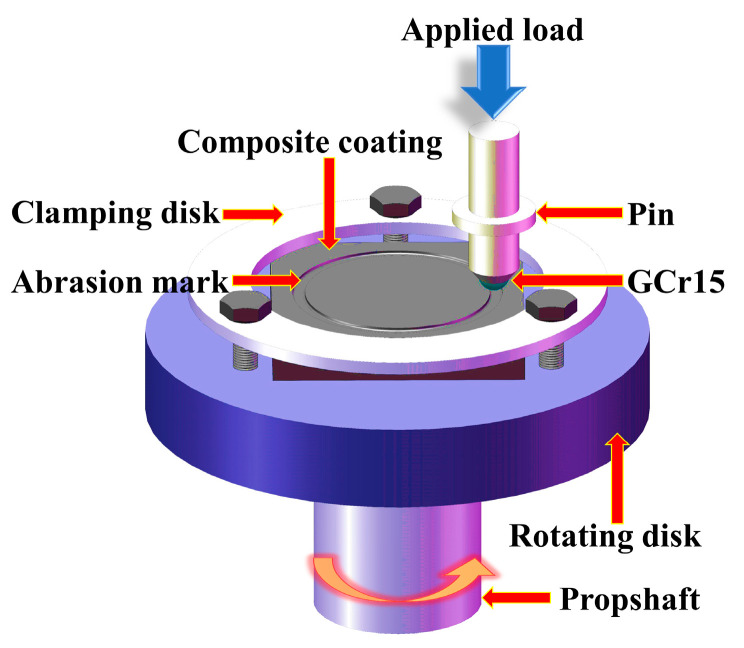
Schematic of friction and wear test details.

The microhardness of the coating sections was measured using a microhardness tester (TIME6610AT, Beijing TIME, China), applying a loading force of 1.96 N for a duration of 15 s. The microhardness of each specimen section was measured three times at the same horizontal position and averaged. The dry wear properties of the composite coatings were tested using a controlled-atmosphere micro-friction and a wear tester (WTM-2E, Lanzhou Zhongke Kaihua, Lanzhou, China). The grinding ball used in the experiment was a GCr15 ball with a diameter of 5 mm. The applied load, sliding speed, and wear time were 7.84 N, 9.42 m/min, and 60 min, respectively. Friction and wear tests for each coating were performed three times under the same conditions. Details of the friction wear test are shown in Figure 2. The friction coefficients were simultaneously obtained using the test system. Following ultrasonic cleaning of the specimens with alcohol both before and after the wear test, the weight loss due to wear was calculated by averaging three measurements using an electronic balance (BSA124S, Sartorius, Göttingen, Germany) with an accuracy of ±0.1 mg. In addition, the abrasion width was measured and averaged four times at different locations for each specimen’s abrasion marks (using scanning electron microscopy).

## 3. Results and Discussion

### 3.1. Phase Analysis

**Figure 3:**
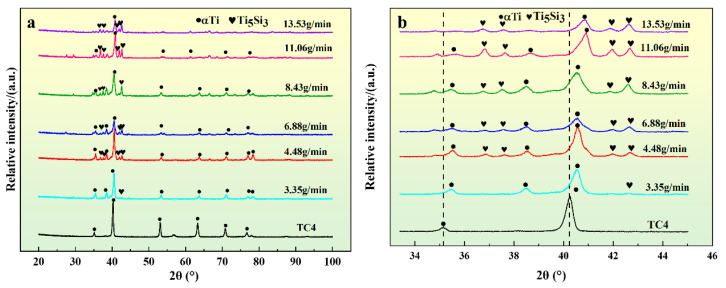
XRD patterns of composite coatings with different powder feeding speeds. (**a**) Complete photograph; (**b**) enlarged photograph.

Figure 3 depicts the XRD patterns of Si-TC4 overlay coatings at various powder feeding rates. It can be seen that the composite coating is mainly composed of α-Ti (PDF#44-1294) and Ti_5_Si_3_ (PDF#29-1362) [36]. The emergence of Ti_5_Si_3_ diffraction peaks in the composite indicates that the presence of the Si element led to a reaction with the Ti element in the molten pool, leading to the formation of Ti_5_Si_3_. It can be inferred that the reaction between Ti and Si in the molten pool leads to the formation of Ti–Si intermetallic compounds represented by Ti_5_Si_3_ [37]. From Figure 3b, it can be seen that the intensities of the α-Ti diffraction peaks decrease, and the intensities of the Ti_5_Si_3_ diffraction peaks increase as more Si elements are added, which is due to more Si elements combining with Ti to form intermetallic compounds. Furthermore, with the addition of Si elements, the diffraction peak of α-Ti shifts to the right, and according to the Bragg diffraction law (Equation (1)), it is known that the crystallite spacing of α-Ti is reduced due to the inclusion of Si elements with a smaller atomic radius.

2dsinθ = nλ(1)

### 3.2. Microstructure Analysis

**Figure 4:**
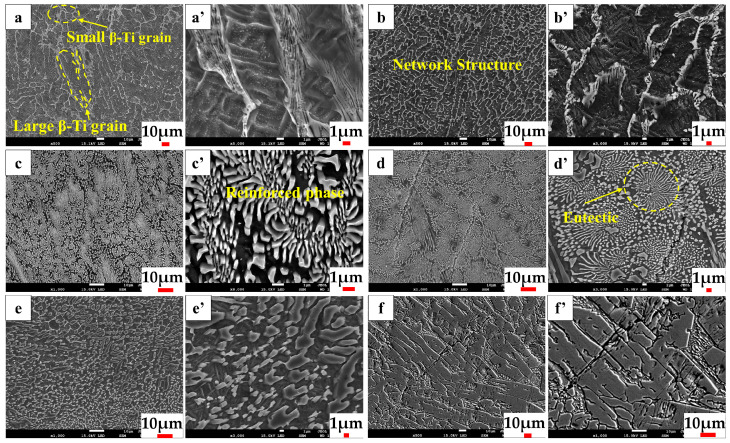
Microstructure of the middle section of the coatings at various powder feeding rates. (**a**,**a’**) 3.35 g/min; (**b**,**b’**) 4.48 g/min; (**c**,**c’**) 6.88 g/min; (**d**,**d’**) 8.43 g/min; (**e**,**e’**) 11.06 g/min; (**f**,**f’**)13.53 g/min.

Figure 4 depicts the microstructure of the middle section of the coatings at various powder feeding rates. The illustration depicts that when powder feeding speeds are lower, a small amount of the reinforced phase in the cladding layer precipitates along the β-Ti grain boundaries [38,39]. As the powder feeding speed rises, there is a continuous increase in the reinforced phase within the cladding layer. When the powder feeding speed reaches 6.88 g/min, the reinforced phase in the cladding layer exhibits a dense distribution with a petal-like morphology. When the powder feeding rate reaches 8.43 g/min, the petal-like reinforced phase in the cladding layer shows a more obvious eutectic structure. When the feeding rate is further increased, the reinforcing phase within the coating transforms into a segmented rod-like morphology with obvious directionality. Finally, when the feeding rate reaches 13.53 g/min, the reinforcing phase becomes plate-like and almost covers the entire coating. However, this also results in an increase in the brittleness of the coating [40].

**Figure 5:**
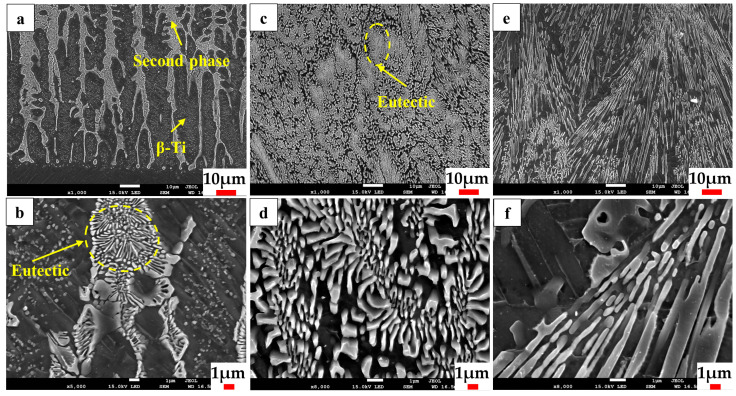
Microstructure of the composite coating feeding rate of 6.88 g/min. (**a**,**b**) Bottom part; (**c**,**d**) middle part; (**e**,**f**) upper part.

Figure 5 depicts the microstructure of the composite coating when the powder feeding rate is 6.88 g/min. Figure 5a,b demonstrates the distinct dendritic crystal morphology at the bottom of the coating, with the dendrites growing upwards perpendicular to the fusion line. The precipitation of the reinforcing phase occurs along the grain boundaries within the coating. Figure 5c,d depicts the microstructure of the middle section of the coating. In the middle of the coating, the reinforcing phase is densely distributed in the form of petal clusters, while the β-Ti phase almost disappears [41]. The petal-like reinforcing phase is formed by a regular cluster of very fine precipitates, with the central precipitates being the smallest in size and the periphery precipitates having a longer lamellar shape. Figure 5e,f illustrates the microstructure of the upper portion of the coating. The reinforcing phases are arranged in the same direction to form a long strip; this is due to a large amount of Si element in the upper part of the melt pool. In the process of crystallization, titanium–silicon intermetallic compounds are the first to precipitate into the incipient phase, and the incipient phase further grows to form a long strip of reinforcing phases.

**Figure 6:**
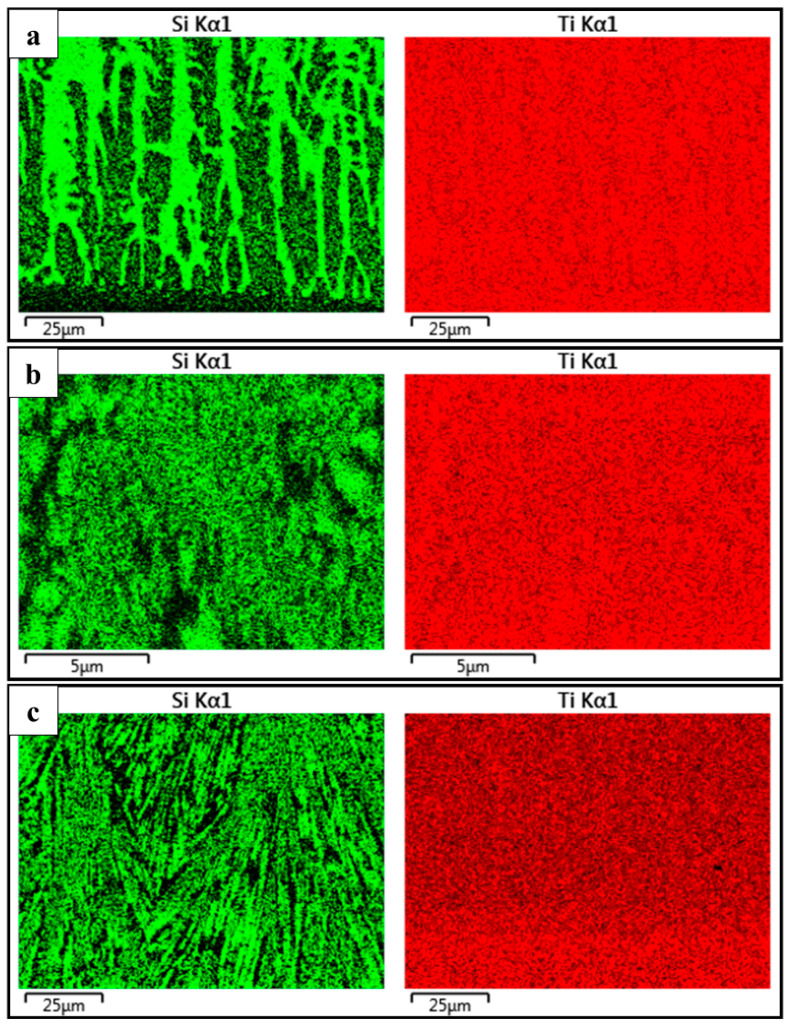
EDS map of the composite coating feeding rate of 6.88 g/min. (**a**) Bottom part; (**b**) middle part; (**c**) upper part.

The EDS results in Figure 6 confirm the presence of large amounts of Ti–Si intermetallic compounds in the composite coating.

**Figure 7:**
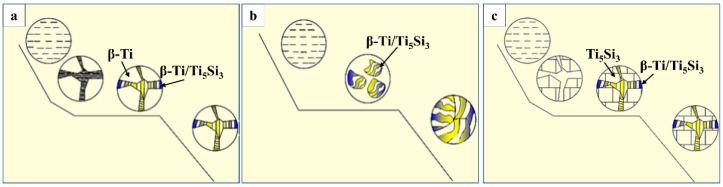
Schematic diagram of the composite coatings’ microstructure formation. (**a**) 4.48 g/min; (**b**) 6.88 g/min; (**c**) 13.53 g/min.

The wire-powder laser cladding coatings exhibit distinct morphological changes as the powder feeding rate varies, transitioning from dendritic formations to petal-like clusters, and ultimately to block-like structures. The changes observed in the microstructure of the cladding layer are attributed to alterations in the crystallization characteristics of the Ti–Si alloy induced by the presence of silicon. When the Si element content is low, the primary phase of β-Ti precipitates from the liquid phase and undergoes slow growth during the cooling process of the melt pool. Consequently, the proportion of Si element in the remaining liquid phase increases relatively. Upon further cooling of the liquid phase, the residual components of the liquid phase reach the eutectic point, leading to the occurrence of a eutectic reaction that results in the formation of β-Ti/Ti_5_Si_3_ [28,31,42]. As the feed rate increases, the proportion of Si in the melt pool continues to increase until it reaches the eutectic point component. As the liquid phase cools, the entire liquid phase undergoes a eutectic reaction to form a petal-like and agglomerated eutectic microstructure (Figure 7b). As the powder feed rate is increased, the Si element content in the melt pool will exceed the eutectic point component. At this stage, the primary phase in the liquid phase will be Ti_5_Si_3_, representing the intermetallic compounds. As the liquid phase undergoes further cooling, incipient phases, such as Ti_5_Si_3_, experience continued growth, leading to a gradual decrease in the relative proportion of the Si element in the remaining liquid phase. When the composition reaches the eutectic point, the residual liquid phase will undergo a eutectic reaction to produce a small amount of β-Ti/Ti_5_Si_3_ eutectic microstructure, which will eventually form a lumpy or rod-like reinforced phase (Figure 7c).

### 3.3. Microhardness and Wear Resistance Analysis

**Figure 8:**
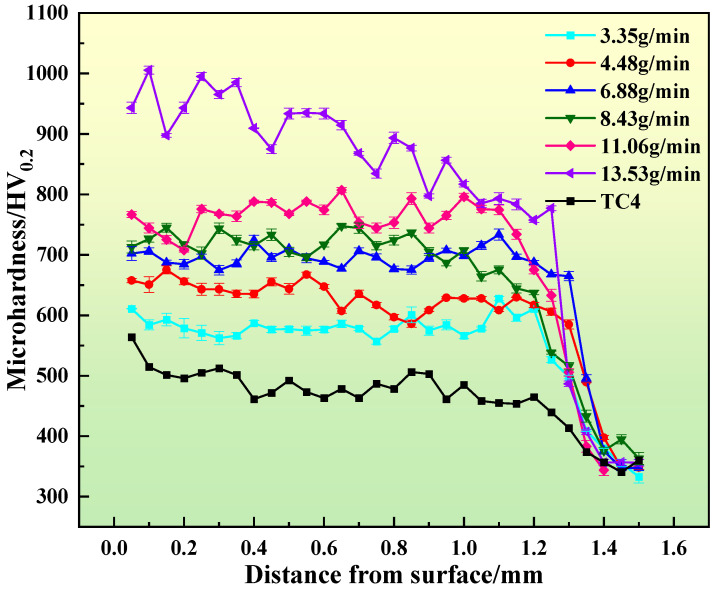
Microhardness of wire-powder laser cladding coating with different powder feeding speeds.

Figure 8 illustrates the distribution of microhardness in the composite coatings during wire-powder laser cladding at different powder feeding speeds. The figure illustrates a stepwise increase in the microhardness of the coating as the powder feed rate increases. The average hardnesses of the composite coatings were 579HV_0.2_, 636HV_0.2_, 694HV_0.2_, 720HV_0.2_, 766HV_0.2_, and 909HV_0.2_, respectively (when the powder feeding rates of Si were 3.35 g/min, 4.48 g/min, 6.88 g/min, 8.43 g/min, 11.06 g/min, and 13.53 g/min, respectively). The increase in the Si element content in the cladding coating results in a corresponding increase in Ti–Si intermetallic compounds within the coating. This leads to a stronger coating produced by the second phase of the strengthening effect, resulting in a gradual increase in the microhardness of the coating. Higher hardness is very favorable for improving the hardness of composite coatings [43,44].

**Figure 9:**
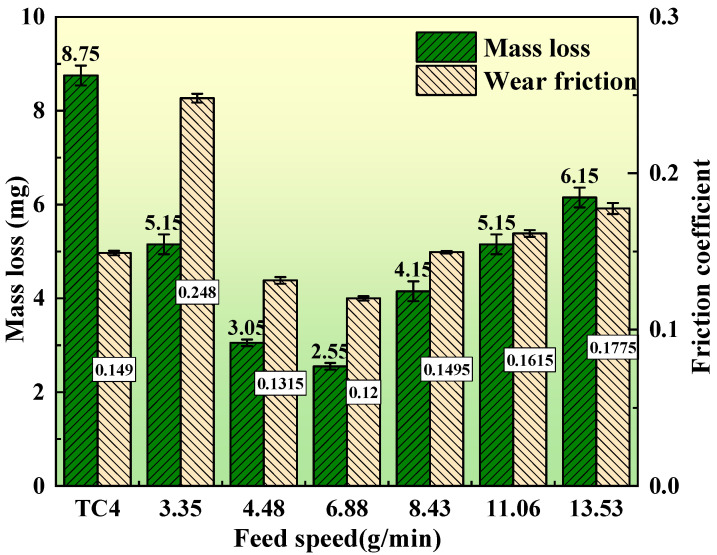
Average friction coefficient and wear weight loss of composite coatings with different powder feeding speeds.

Figure 9 shows the plots of the average friction coefficient and wear weight loss at various feed rates. The plots indicate a decreasing trend, followed by an increasing trend in the average friction coefficient and wear weight loss of the composite coatings. The tribological properties of the composite coatings improve and then deteriorate as the Si content increases. The lowest wear loss (2.55 mg) and coefficient of friction (0.12) and the best tribological performance of the composite coating were obtained when the Si powder was fed at a rate of 6.88 g/min. An optimal content of the Ti–Si-reinforced phase in the coatings exists, leading to the coatings’ superior tribological performance. It can be seen that the change rule of tribological properties of composite coatings is not consistent with the hardness. This is related to the content and morphology of the second phase of Ti–Si; with the increase in the addition of Si powder, the second phase of Ti–Si increases, and the hardness of the composite coating increases. However, only the Ti–Si second phase near the eutectic point composition can significantly improve the tribological properties. The composite coatings with an inappropriate amount of Ti–Si second phase are prone to severe abrasive wear during frictional wear, and this harmful effect will offset or even exceed the beneficial effect of the second phase. It is noteworthy that the coefficient of friction of the composite coatings becomes larger than that of the TC4 coating when a small amount of Si is added (3.35 g/min). This is because, although the addition of a small amount of Si leads to the in situ generation of a small amount of reinforcing phase in the composite coating, this beneficial effect cannot counteract the detrimental effects of its abrasive wear. For similar reasons, the addition of excessively high levels of Si also causes an increase in the coefficient of friction. It can be seen that the control of the Si content is very important for the friction reduction effect. In this study, the addition of Si content close to the eutectic composition (4.48 g/min and 6.88 g/min) was able to reduce friction through the most beneficial second phase enhancement [26,27,30].

**Figure 10:**
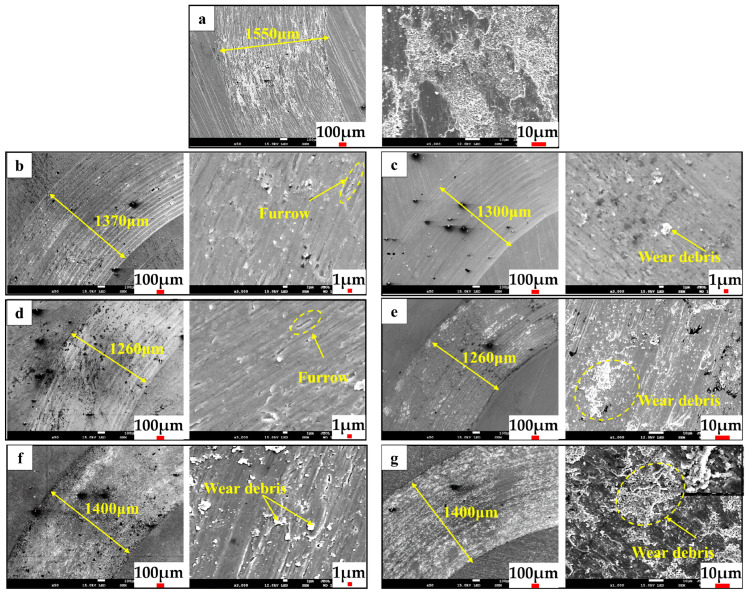
Wear morphology of coatings with different powder feeding speeds. (**a**) TC4 wire cladding coating; (**b**) 3.35 g/min; (**c**) 4.48 g/min; (**d**) 6.88 g/min; (**e**) 8.43 g/min; (**f**) 11.06 g/min; (**g**) 13.53 g/min.

Figure 10 illustrates the morphology of the wear tracks for both the TC4 wire material cladding coating and the wire-powder composite coatings. The abrasion mark widths of the Si–Ti composite coatings exhibit a reduction, compared to the cladding coating of TC4. Furthermore, the abrasion mark widths demonstrate a pattern of decreasing and then increasing, which coincides with the trend of mass loss of the coatings. The TC4 wire cladding coating exhibits a plastic deformation area in the presence of a large abrasion area, whereas the composite coating eliminates the abrasion area. This change in the wear mechanism is associated with the microhardness of the coating [44,45]. The TC4 coating exhibits low microhardness and is susceptible to adhesive wear, whereas the composite coating demonstrates relatively high microhardness and is resistant to adhesive wear. The abrasive scar morphology of the coating shows more obvious furrows and detachment pits, and the furrows in the coating are short and intermittent, with obvious abrasive wear [12,46] phenomenon when the powder feed rate is lower than 8.43 g/min. As the Si content increases, there is a corresponding increase in the hardness of the coating, as well as an increase in brittleness. The white abrasive chips in the coating increase significantly when the powder feed rate is increased above 8.43 g/min. This coincides with the increasing trend of wear weight loss. The formation of intermittent short furrows in the coating is closely related to the shedding and sliding of hard second-phase particles within the coating. During the relative movement of the friction vise, the hard second-phase particles and the abrasive ball will experience multiple collisions and detachment. The dislodgement of hard second-phase particles in the friction vise can result in sliding on the surrounding soft substrate, leading to the generation of a plowing effect. Moreover, the dislodged abrasive chips will collide with the hard second-phase particles in the coating that have remained intact throughout the plowing process, leading to the interruption of plowing. After multiple collisions, the unremoved hard second-phase particles in the coating will be dislodged, leading to the formation of intermittent, short-plowing furrows through repeated occurrences. It can be seen that the wear mechanisms of the composite coating include adhesive and abrasive wear, with limited improvement in tribological performance when the added content of Si powder is low (3.35 g/min) and when the addition content of Si powder is too much (more than 6.88 g/min). With too much Ti–Si second mutual phase collision shedding, abrasive grain wear is more serious, and the friction reduction performance is not outstanding, although the wear resistance is very good. Only when the Ti–Si content is close to the eutectic point composition that the wear mechanism of the composite coating shows only slight abrasive wear, with both excellent wear resistance and friction reduction performance.

## 4. Conclusions

In this paper, Si-TC4 composite coatings were prepared on the surface of a TC4 titanium alloy using the process of high-speed wire-powder laser cladding, and the physical structure, microstructure, chemical composition, microhardness, and wear resistance properties of the coatings were characterized and investigated; the main conclusions are as follows:

(1) The composite coatings are mainly composed of intermetallic compounds, such as αTi and Ti_5_Si_3_. With the increase in Si content, the Ti–Si intermetallic compounds in the composite coatings increase, and the grains become finer.
(2) As the Si content increases, the solidification and crystallization process of the composite coatings change, showing the microstructure changes in Ti–Si from sub-eutectic to eutectic and per-eutectic, resulting in a microstructure showing a regular transformation process, such as granular, eutectic clusters, stripes, and rods, as well as blocks.
(3) As the Si content increases, the microhardness of the composite coatings gradually increases, and the wear resistance shows a tendency to improve and then deteriorate, due to the intermetallic compounds, such as Ti_5_Si_3_, formed in situ in the coatings with different morphologies. The friction coefficients of the composite coatings with different Si contents were changed, due to the competition between the favorable factor of enhancement and the unfavorable factor of abrasive wear brought about by the in situ generation of the second phase at the same time. The addition of less or more Si content leads to an increase in the coating friction coefficient. The best tribological properties were obtained for the composite coating with the eutectic structure dominated by the powder feed rate of 6.88 g/min.
(4) The wear mechanism of the TC4 wire coating threads was adhesive wear, and that of the Si-TC4 composite coatings was mainly abrasive wear, which was significantly influenced by the addition of Si and the presence of intermetals like Ti_5_Si_3_.
(5) According to the research results of this paper, the next step of in-depth research should focus on the precise control of the chemical composition, temperature, organization, and properties of Si-TC4 composite coatings prepared by high-speed wire-powder laser cladding.

## Figures and Tables

**Table 1:** Chemical composition of TC4 wire (wt.%).

Fe	C	N	H	O	Al	V	Ti
≤0.30	≤0.10	≤0.05	≤0.015	≤0.20	5.5–6.8	3.5–4.5	Bal.

**Table 2:** Process parameters of high-speed laser cladding.

Power/W	Scanning Speed/mm∙s^−1^	Wire-Feeding Speed/mm∙s^−1^	Hot Wire Coefficient	Protective Gas Flow/L∙min	Spot Size/mm	Overlap Ratio/%
2000	30	20	1.10	15	3	30

## Data Availability

No data were used for the research described in the article.

## References

[1] Pathania A., Anand Kumar S., Nagesha B.K., Barad S., Suresh T.N. (2021). Reclamation of titanium alloy based aerospace parts using laser based metal deposition methodology. Mater. Today Proc..

[2] Li Z., Takano N., Mizutani M. (2023). Material properties of selective laser melting additive-manufactured Ti6Al4V alloys with different porosities. Precis. Eng..

[3] Hatem A., Lin J., Wei R., Torres R.D., Laurindo C., de Souza G.B., Soares P. (2018). Tribocorrosion behavior of low friction TiSiCN nanocomposite coatings deposited on titanium alloy for biomedical applications. Surf. Coat. Technol..

[4] June D., Mayeur J.R., Gradl P., Wessman A., Hazeli K. (2024). Effects of size, geometry, and testing temperature on additively manufactured Ti-6Al-4V titanium alloy. Addit. Manuf..

[5] Pede D., Li M., Virovac L., Poleske T., Balle F., Müller C., Mozaffari-Jovein H. (2022). Microstructure and corrosion resistance of novel β-type titanium alloys manufactured by selective laser melting. J. Mater. Res. Technol..

[6] Takesue S., Kikuchi S., Akebono H., Misaka Y., Komotori J. (2019). Effect of pre-treatment with fine particle peening on surface properties and wear resistance of gas blow induction heating nitrided titanium alloy. Surf. Coat. Technol..

[7] Murmu A.M., Parida S.K., Das A.K., Kumar S. (2023). Evaluation of laser cladding of Ti6Al4V-ZrO_2_-CeO_2_ composite coating on Ti6Al4V alloy substrate. Surf. Coat. Technol..

[8] Chirico C., Romero A.V., Gordo E., Tsipas S.A. (2022). Improvement of wear resistance of low-cost powder metallurgy β-titanium alloys for biomedical applications. Surf. Coat. Technol..

[9] Park C.W., Adomako N.K., Lee M.G., Kim J.H., Kim J.H. (2021). Interfacial structure and pore formation mechanism during laser cladding of pure vanadium on Ti-6Al-4V alloy. Int. J. Refract. Met. Hard Mater..

[10] Okoli U.O., Otunniyi I.O., Adebiyi I.D. (2021). Dry sliding wear behaviour of binary powder laser cladded Ti-6Al-4V using SiC and Al. Mater. Today Proc..

[11] Kang N., El Mansori M., Feng E., Zhao C., Zhao Y., Lin X. (2022). Sliding wear and induced-microstructure of Ti-6Al-4V alloys: Effect of additive laser technology. Tribol. Int..

[12] Ko U.J., Jung J.H., Kang J.H., Choi K., Kim J.H. (2024). Enhanced microstructure and wear resistance of Ti–6Al–4V alloy with vanadium carbide coating via directed energy deposition. Materials.

[13] Han B., Zhang S., Zhang T., Chen Y., Qin X., Li M., Hu C., Wei M., Xue X. (2023). Hardness enhancement mechanism of Al_x_CoCrFeNiSi high-entropy alloy coatings prepared by laser cladding. Intermetallics.

[14] Zhang Q., Wang Q., Han B., Li M., Hu C., Wang J. (2023). Comparative studies on microstructure and properties of CoCrFeMnNi high entropy alloy coatings fabricated by high-speed laser cladding and normal laser cladding. J. Alloys Compd..

[15] Li S., Yamaguchi T. (2022). High-temperature oxidation performance of laser-cladded amorphous TiNiSiCrCoAl high-entropy alloy coating on Ti-6Al-4V surface. Surf. Coat. Technol..

[16] Dey D., Bal K.S., Khan I., Bangia I., Singh A.K., Roy Choudhury A. (2022). Study of tribo-mechanical properties of laser clad Al_2_O_3_-TiB_2_-TiN-BN‖Ti-6Al-4V alloy. Opt. Laser Technol..

[17] Verdi D., Cortés R., Chia G.Y., Tay G. (2024). Erosion behaviour of laser cladded Inconel 625—Vanadium carbide metal matrix composites coatings manufactured with different reinforcement contents. Surf. Coat. Technol..

[18] Schopphoven T., Gasser A., Wissenbach K., Poprawe R. (2016). Investigations on ultra-high-speed laser material deposition as alternative for hard chrome plating and thermal spraying. J. Laser Appl..

[19] Abuabiah M., Mbodj N.G., Shaqour B., Herzallah L., Juaidi A., Abdallah R., Plapper P. (2023). Advancements in Laser Wire-Feed Metal Additive Manufacturing: A Brief Review. Materials.

[20] Kotar M., Fujishima M., Levy G.N., Govekar E. (2021). Advances in the understanding of the annular laser beam wire cladding process. J. Mater. Process. Technol..

[21] Gao L., Chuang A.C., Kenesei P., Ren Z., Balderson L., Sun T. (2024). An operando synchrotron study on the effect of wire melting state on solidification microstructures of Inconel 718 in wire-laser directed energy deposition. Int. J. Mach. Tools Manuf..

[22] Singh S.N., Deoghare A.B. (2023). Microstructure, micro-hardness and tensile properties of Ti6Al4V manufactured by high layer-thickness wire-feed multi-laser directed energy deposition. Mater. Lett..

[23] Wang C., Suder W., Ding J., Williams S. (2021). Wire based plasma arc and laser hybrid additive manufacture of Ti-6Al-4V. J. Mater. Process. Technol..

[24] Wang L.X., Huang Y.M., Jia C.P., Yang L.J., Yan S. (2023). Laser-directed energy deposition of in-situ titanium-matrix coatings with a Ti-B4C cored wire. Addit. Manuf..

[25] Wang F., Mei J., Wu X. (2006). Microstructure study of direct laser fabricated Ti alloys using powder and wire. Appl. Surf. Sci..

[26] Farayibi P.K., Murray J.W., Huang L., Boud F., Kinnell P.K., Clare A.T. (2014). Erosion resistance of laser clad Ti-6Al-4V/WC composite for waterjet tooling. J. Mater. Process. Technol..

[27] Hua Z., Xiong L., Zhang M., Wang C., Mi G., Jiang P. (2023). Microstructure evolution and tribological properties of (TiB+TiC)/Ti–6Al–4V composites fabricated via in situ laser-directed energy deposition of wire and powders in an underwater environment. Compos. B Eng..

[28] Zhuo L., Ji K., Lu J., Sun J., Huo W., Shao H., Chen B., Zhao Y. (2023). Microstructure characterization and tensile performance of a high-strength titanium alloy with in-situ precipitates of Ti_5_Si_3_. J. Alloys Compd..

[29] Gu D., Hagedorn Y.-C., Meiners W., Wissenbach K., Poprawe R. (2011). Selective Laser Melting of in-situ TiC/Ti_5_Si_3_ composites with novel reinforcement architecture and elevated performance. Surf. Coat. Technol..

[30] Zhao X., Lyu P., Fang S., Li S., Tu X., Ren P., Liu D., Chen L., Xiao L., Liu S. (2024). Microstructure and Wear Behavior of Ti-xFe-SiC In Situ Composite Ceramic Coatings on TC4 Substrate from Laser Cladding. Materials.

[31] Yan H., Liu K., Zhang P., Zhao J., Qin Y., Lu Q., Yu Z. (2020). Fabrication and tribological behaviors of Ti_3_SiC_2_/Ti_5_Si_3_/TiC/Ni-based composite coatings by laser cladding for self-lubricating applications. Opt. Laser Technol..

[32] Zhou Z.-Y., Liu X.-B., Zhuang S.-G., Wang M., Luo Y.-S., Tu R., Zhou S.-F. (2019). Laser in-situ synthesizing Ti_5_Si_3_/Al_3_Ni_2_ reinforced Al_3_Ti/NiTi composite coatings: Microstructure, mechanical characteristics and oxidationbehavior. Opt. Laser Technol..

[33] Cui J., Lin C., Peng X., Yang J., Ren T., Ma Q., Li F., Shi Y., Huang S., Yin G. (2024). Phase transition and atomic competition mechanism of in-situ particle reinforced Ti_x_Al/Ti_5_Si_3_ composite coating prepared by laser cladding Al-xSi-2Nb alloy powder on Ti6Al4V alloy. Surf. Coat. Technol..

[34] Huang X., Gao Y., Li Q., Jian Y., Xiao P., Li B., Wang Y., Yi Y., Zhao S. (2021). Effect of Si element on improving the oxidation resistance of hybrid (Ti_5_Si_3_ + TiC) particles reinforced Ti6Al4V matrix composites. Corros. Sci..

[35] Su W., Cui X., Yang Y., Guan Y., Zhao Y., Wan S., Li J., Jin G. (2021). Effect of Si content on microstructure and tribological properties of Ti_5_Si_3_/TiC reinforced NiTi laser cladding coatings. Surf. Coat. Technol..

[36] Lu Q., Lv Y., Zhang C., Zhang H., Chen W., Xu Z., Feng P., Fan J. (2022). Highly oxidation-resistant Ti-Mo alloy with two-scale network Ti_5_Si_3_ reinforcement. J. Mater. Sci. Technol..

[37] Mathabathe M.N., Bolokang A.S., Govender G., Mostert R.J., Siyasiya C.W. (2018). Structure-property orientation relationship of a γ/α_2_/Ti_5_Si_3_ in as-cast Ti-45Al-2Nb-0.7Cr-0.3Si intermetallic alloy. J. Alloys Compd..

[38] Caglar H., Liang A., Groom K., Mumtaz K. (2024). Multi-laser powder bed fusion of Ti6Al4V: Diode area melting utilizing low-power 450 nm diode lasers. J. Mater. Process. Technol..

[39] Kaoushik V.M., Nichul U., Chavan V., Hiwarkar V. (2023). Development of microstructure and high hardness of Ti6Al4V alloy fabricated using laser beam powder bed fusion: A novel sub-transus heat treatment approach. J. Alloys Compd..

[40] Önder S., Saklakoğlu N., Sever A. (2023). Selective laser melting of Ti6Al4V alloy: Effect of post-processing on fatigue life, residual stress, microstructure, microhardness and surface roughness. Mater. Charact..

[41] Dareh Baghi A., Nafisi S., Ebendorff-Heidepriem H., Ghomashchi R. (2022). Microstructural Development of Ti-6Al-4V Alloy via Powder Metallurgy and Laser Powder Bed Fusion. Metals.

[42] Tao C., Li L., He N., Sun G., Liu C., Xu J., Li M., Dong L., Zhang Y., Wang L. (2023). Microstructure and mechanical properties of in-situ Ti_5_Si_3_/TC4 composites via spark plasma sintering and hot rolling. J. Alloys Compd..

[43] Al-Sayed Ali S.R., Hussein A.H.A., Nofal A.A.M.S., Hasseb Elnaby S.E.I., Elgazzar H.A., Sabour H.A. (2017). Laser powder cladding of Ti-6Al-4V α/β alloy. Materials.

[44] Razumov N., Masaylo D., Kovalev M., Volokitina E., Mazeeva A., Popovich A. (2023). Structure and wear resistance of composite TiC-NiMo coating produced by L-DED on Ti-6Al-4V substrate. Metals.

[45] Kushwaha A., Subramaniyan A.K., Bommanahalli Kenchappa N., Barad S. (2022). Microstructure, mechanical, and wear properties of thin-walled Ti6Al4V parts produced using laser powder bed fusion technique. Mater. Lett..

[46] Obadele B.A., Andrews A., Olubambi P.A., Mathew M.T., Pityana S. (2015). Effect of ZrO_2_ addition on the dry sliding wear behavior of laser clad Ti6Al4V alloy. Wear.

